# Imaging Sequences for Hyperpolarized Solids

**DOI:** 10.3390/molecules26010133

**Published:** 2020-12-30

**Authors:** Xudong Lv, Jeffrey Walton, Emanuel Druga, Raffi Nazaryan, Haiyan Mao, Alexander Pines, Ashok Ajoy, Jeffrey Reimer

**Affiliations:** 1 Department of Chemistry, University of California, Berkeley, CA 94720, USA; david.lv@berkeley.edu (X.L.); epieon@berkeley.edu (E.D.); rnazaryan@berkeley.edu (R.N.); pines@berkeley.edu (A.P.); ashokaj@berkeley.edu (A.A.); 2 Nuclear Magnetic Resonance Facility, University of California Davis, Davis, CA 95616, USA; jhwalton@ucdavis.edu; 3 Department of Chemical and Biomolecular Engineering, University of California, Berkeley, CA 94720, USA; maohaiyan@berkeley.edu; 4 Lawrence Berkeley National Laboratory, Materials Science Division, University of California, Berkeley, CA 94720, USA

**Keywords:** hyperpolarization, magnetic resonance imaging, flip angle

## Abstract

Hyperpolarization is one of the approaches to enhance Nuclear Magnetic Resonance (NMR) and Magnetic Resonance Imaging (MRI) signal by increasing the population difference between the nuclear spin states. Imaging hyperpolarized solids opens up extensive possibilities, yet is challenging to perform. The highly populated state is normally not replenishable to the initial polarization level by spin-lattice relaxation, which regular MRI sequences rely on. This makes it necessary to carefully “budget” the polarization to optimize the image quality. In this paper, we present a theoretical framework to address such challenge under the assumption of either variable flip angles or a constant flip angle. In addition, we analyze the gradient arrangement to perform fast imaging to overcome intrinsic short decoherence in solids. Hyperpolarized diamonds imaging is demonstrated as a prototypical platform to test the theory.

## 1. Introduction

NMR is central to many chemical, biological and material analysis due to the rich chemical information it can provide [1,2]. MRI, as the imaging counter part of NMR, is a powerful tool in medicine and biology [3,4]. However, the sensitivity of both techniques relies on nuclear spin polarization, which is intrinsically low at thermal equilibrium. One compelling approach to tackle this insensitivity is hyperpolarization. This approach brings the nuclear spin polarization level beyond thermal equilibrium to produce many orders of magnitude higher signal. Routes to hyperpolarization includes dynamic nuclear polarization (DNP) [5], parahydrogen induced hyperpolarization (PHIP) [6], as well as chemically-induced DNP (CIDNP) [7]. While the methods of hyperpolarization can be applied in both liquids and solids, hyperpolarized solids are particularly attractive as an imaging agent in nano-medicine [8], or as a polarization hub to deliver hyperpolarization for general chemicals [9]. However, challenges remain on how to image hyperpolarized solids given the none-replenishable nature of the polarization and short coherence times of solids.

**Figure 1:**
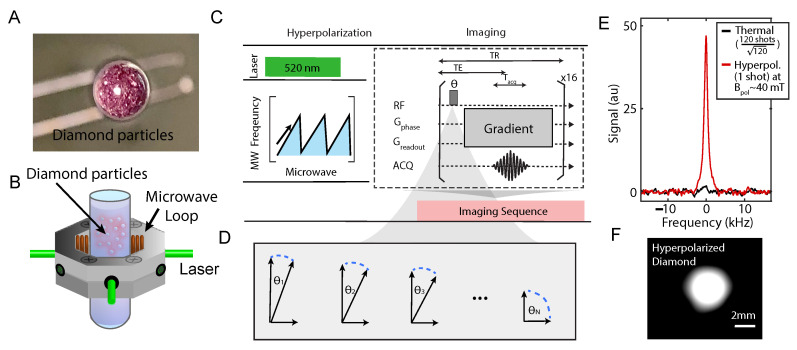
Experiment schematic. (**A**) A picture of diamond particles (~200 μm in size) contained in an NMR tube as an imaging phantom (taken from the bottom of a NMR tube). (**B**) Green laser excitation and MW irradiation is applied on the sample in order to transferred polarization to lattice ${}^{13}$C nuclei from optically polarized NV- electrons in the microscopic scale. (**C**) Experimental protocol of hyperpolarizing and imaging diamonds. ${}^{13}$C hyperpolarization occurs at 38 mT under MW sweeps across the NV-ESR spectrum, and then transferred to a MRI machine for imaging. Flip angles and gradient arrangement determine the quality of the MRI. (**D**) Illustration of flip angles for the *n*th repetitions. (**E**) Typical signal enhancement by hyperpolarization, showing signal gain against signal at 7 T. For a fair comparison, the noise in both is normalized to be 1 (dash line). (**F**) A typical MR image of diamond phantom in (**A**).

In the work, we use diamond particles (Figure 1A) as a prototypical platform to test the imaging sequences (Figure 1C,D) and to provide some theoretical understanding of the results as well as some insight into sequence design for imaging similar hyperpolarized materials. The hyperpolarization in diamond is enabled by one type of special atom-like defect—the Nitrogen Vacancy (NV) center [10] and a recently developed protocol [9,11]. The electronic spins of NV centers are optically polarizable to ≈99% at room temperature [10], and their long coherence time ensures its efficiency at polarizing surrounding ${}^{13}$C nuclear spins via chirped MW (Figure 1B,C). ${}^{13}$C imaging of natural abundance diamond powders (Figure 1F) is only possible with such highly polarized signal (Figure 1E). The ability to image micron/nanodiamonds through MRI can open up possibilities in directions including physics, chemical and biological analysis. For instance, hyperpolarized diamond particles that “light up” in MRI mode can potentially be applied as a targeting and tracking agent given their bio-compatibility and surface modifiability [11,12,13,14]. Additional advantage of high surface-to-volume ratio can also enable polarization transfer to external nuclei when brought into close contact with other chemicals for high-SNR and high-resolution NMR [15].

The analysis of the imaging sequence for a diamond prototypical system relies on a theoretical framework we develop herein for imaging hyperpolarized solids in general. The theoretical framework considers two major components of an MRI sequence—flip angle and gradients (Figure 1C), which determine the quality of an MR image.

In an MRI sequence, a radio frequency pulse is normally applied at the beginning of each repetition, in order to rotate the magnetization from z direction to the xy plane, so that the nuclear Larmor precession can be detected. The angle of such rotation is referred as flip angle. In conventional MRI without hyperpolarization, the z magnetization can be recovered after each repetition by the $T_{1}$ relaxation process. In contrast, for the cases of hyperpolarization, the initial polarization is much higher beyond the equilibrium state; thus, relaxation tends to reduce it towards a much lower level. As a results, some sequence design principles in conventional MRI no long hold in such cases, and it requires careful engineering of flip angles to be suited for imaging hyperpolarized objects. The high level of the magnetization, if effectively distributed, can enhance the image SNR and resolution by orders of magnitude.

Not only does flip angle have to be designed uniquely for hyperpolarized solid state imaging, better arrangement of the gradient and pulses are critical as well. As a result of the nature of solids, static coupling between nuclei leads to short coherence times. This suggests that one has to either perform imaging rapidly or apply pulse sequences to protect coherence. We present strategies that either facilitate fast imaging or refocus signals by decoupling sequences with a focus of ${}^{13}$C MRI in diamonds.

## 2. Results

### 2.1. Image Equation

We analyze the dynamic of the magnetization change under certain flip angle pulses and theoretically present optimal solutions. In this section, we consider two major scenarios, i.e. dynamically changeable flip angles and a constant flip angle over different repetitions. We also consider two metrics for our optimization – total magnetization, which corresponds to total signal of the image, and the uniformity of the signal across repetitions.

More explicitly, we write down the signal equation of an MR image in terms of the xy plane magnetization $M_{x}$ [16]:

(1) $$S\left(k_{x},k_{y}\right)=\;\int \int \;M_{x}\left(x,y,k_{x},k_{y}\right)e^{-i2\pi \left(k_{x}x+k_{y}y\right)}e^{-t\left(k_{x},k_{y}\right)/T_{2}^{*}}dxdy$$

where $k_{x}=\gamma /2\pi \int_{0}^{t}G_{x}\left(t\right)\mathrm{dt}$, $k_{y}=\gamma /2\pi \int_{0}^{t}G_{y}\left(t\right)\mathrm{dt}$ (γ is the gyromagnetic ratio, and $G_{x}$, $G_{y}$ are gradients along x and y axis). Note that this signal equation takes into consideration that the transverse magnetization $M_{x}$ as a function of $k_{x}$ and $k_{y}$ can be different for each repetition. This dependence can be expressed as:

(2) $$M_{x}\left(x,y,k_{x},k_{y}\right)=K\left(k_{x},k_{y}\right)\cdot \rho \left(x,y\right)$$

where $K(k_{x},k_{y})$ (we refer as *magnetization factor*) is the factor representing non-uniform excitation in each repetitions (for instance a progression of small tip angle pulses), and ρ(x,y) is the nuclear spin density at location (x,y). Performing a Fourier transform of $S\left(k_{x},k_{y}\right)$, we obtain the image represented in the real space:

(3) $$I\left(x,y\right)=F\left(K\right)*F\left(e^{-t\left(k_{x},k_{y}\right)/T_{2}^{*}}\right)*\rho \left(x,y\right)$$

where F represents Fourier transformation and ∗ represents convolution. Note that by taking the limit of $t<<T_{2}^{*}$, and assuming uniform excitation cross different repetition, the Equation (3) reduces to I(x,y)=ρ(x,y).

The image equation (Equation (3)) is different from a typical image equation as the first term represents the effect of flip angles, which is special to the case of hyperpolarization. In repetition *n*, we denote this effect to be $K_{n}$. In the case of Cartesian sampling, we can write $n=k_{x}$ without losing generality.

### 2.2. Flip Angle Consideration

How does $K_{n}$ depend on the flip angle θ? We address this question by considering two cases: dynamically changing flip angles and a constant flip angle. In practice, whether one has the ability to program the flip angle for each repetition on the MRI machine determines which case will be utilized.

Variable flip angle—First we consider the most general scenario where one has control on the flip angle of each repetition. This stems from an intuitive demand that magnetization remains same in each repetition, similar to the magnetization in saturation recovery sequences. More specifically, if we implement an imaging sequence with a repetition time TR to a nuclear spin system with relaxation time $T_{1}$ and an equilibrium magnetization $M_{0}$, we can write the dynamic equation as following [17]:

(4) $$\left\{\begin{array}{c} M_{n}=\left(M_{n-1}\cos\theta_{n-1}-M_{0}\right)e^{-\frac{TR}{T_{1}}}+M_{0}\\ M_{x,n}=M_{n}\sin\theta_{n} \end{array}\right.$$

where we denote in the *n*th repetition, the flip angle to be $\theta_{n}$, the longitudinal and transverse magnetization to be $M_{n}$ and $M_{x,n}$ respectively. Given that magnetization can be written as multiplication of the magnetization factor and the spin density: $M_{n}=K_{n}*\rho \left(x,y\right)$, $M_{x,n}=K_{x,n}*\rho \left(x,y\right)$, and $M_{0}=K_{0}*\rho \left(x,y\right)$, we can eliminate the location information in ρ(x,y), and simplify the dynamic equation in terms of magnetization factor *K*.

(5) $$\left\{\begin{array}{c} K_{n}=\left(K_{n-1}\cos\theta_{n-1}-K_{0}\right)e^{-\frac{TR}{T_{1}}}+K_{0}\\ K_{x,n}=K_{n}\sin\theta_{n} \end{array}\right.$$

The initial magnetization factor in the hyperpolarization case is $K_{\mathrm{hp}}$, in contrast to the thermal polarization case $K_{0}$. With such initial condition, we solve the recurrent dynamic equation (Equation (5)) and obtain:

(6) $$K_{x,n}=K_{\mathrm{hp}}\left[\prod_{k=1}^{n-1}\left(\Gamma \cos\theta_{k}\right)+\frac{1}{K_{\mathrm{hp}}/K_{0}}\left(1-\Gamma \right)\times \left\{\sum_{i=2}^{n}\prod_{k=i}^{n-1}\left(\Gamma \cos\theta_{k}\right)\right\}\right]\sin\theta_{n}$$

where $\Gamma =e^{-\frac{TR}{T_{1}}}$. We assume hyperpolarization enhances signal much higher than thermal signal, suggesting $K_{\mathrm{hp}}>>K_{0}$. With such approximation, we have the leading order $K_{x,n}=K_{\mathrm{hp}}\left[\prod_{k=1}^{n-1}\left(\Gamma \cos\theta_{k}\right)\right]\sin\theta_{n}$.

One of the advantages of having ability to dynamically varying the flip angle is that the transverse magnetization $M_{x}$ in each repetition can be constant by carefully design the flip angles. This allows one to avoid image distortion along the phase encoding direction (further detailed in the Discussion section). Applying the condition of $K_{x,n}=\;\mathrm{constant}\;$, we can obtain (see Appendix A.1):

(7) $$\tan^{2}\theta_{n}=\left(1-\Gamma^{3}\right)\cdot \frac{\Gamma^{2N-2n-1}}{1-\Gamma^{2N-2n-1}}$$

where *N* is the total number of repetitions.

**Figure 2:**
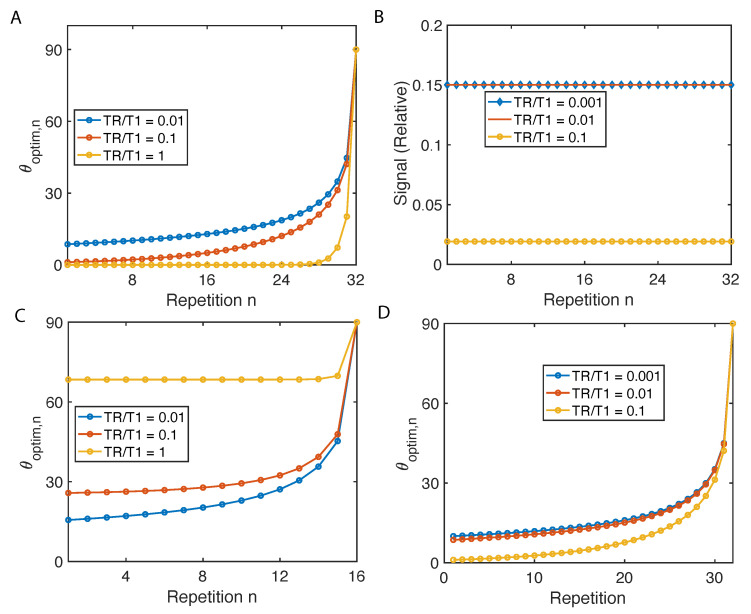
Variable flip angles for constant signal and maximum signal. (**A**) The flip angles to enable constant magnetization in a 32 repetition imaging sequence are determined based on Equation (7). (**B**) Implementing flip angles in (**A**), the relative transverse magnetization signal is simulated taking $K_{\mathrm{hp}}=1$ and $K_{0}=10^{-3}$. (**C**,**D**) The flip angles to maximize the cumulative signal under different TR/T1.

As shown in Figure 2A, flip angles have to increase with the number of repetitions in order to maintain same transverse magnetization, and all three curves with different TR/T1 converge to $90^{\circ }$ to saturate all the magnetization. As a result, the relative transverse magnetization stays flat throughout the scan confirmed by simulation (see Figure 2B). Such uniform magnetization factor allows $K(k_{x},k_{y})$ to be constant, resulting in F(K) to be a delta function, and the reconstructed image I(x,y) in Equation (3) to be: $I\left(x,y\right)\propto F\left(e^{-t\left(k_{x},k_{y}\right)/T_{2}^{*}}\right)*\rho \left(x,y\right)$, immune from image blur cased by excitation.

In addition to constant magnetization, one desires to gain as large cumulative signal as possible, which leads to a different optimization problem.

(8) $${\arg\max}_{\theta_{n}}\left\{S_{\mathrm{cumulative}\;}=\sum_{n=1}^{N}M_{x,n}\right\}$$

If $\theta_{N}$ is optimal, it should satisfy: $\frac{\partial S_{\mathrm{cumulative}}}{\partial \theta_{N}}=\Gamma^{N-1}\cos\theta_{1}\cdots \cos\theta_{N-1}\cos\theta_{N}=0$.

Similarly, we can get:

(9) $$\begin{array}{c} \frac{\partial S_{\mathrm{cumulative}}}{\partial \theta_{N-1}}=\Gamma^{N-1}\cos\theta_{1}\cdots \left(-\sin\theta_{N-1}\right)\sin\theta_{N}+\Gamma^{N-2}\cos\theta_{1}\cdots \cos\theta_{N-1}=0\\ \Rightarrow \Gamma \sin\theta_{N-1}\sin\theta_{N}=\cos\theta_{N-1} \end{array}$$

In general, the relationship between two consecutive flip angles is: $\sin\theta_{n+1}=\Gamma \tan\theta_{n}$. Iteratively solving this sequence from the end where $\sin\theta_{N}=1$ (see Appendix A.2), we have:

(10) $$\theta_{n}=\tan^{-1}\sqrt{\frac{1}{\Gamma^{2}}\cdot \frac{1-\Gamma^{2}}{1-\Gamma^{2(N-n)}}}$$

And such results of N=16 and 32 are presented in Figure 2C,D.

So far, we have derived the design principle of variable flip angle pulses to achieve either constant magnetization or maximum total magnetization. We here briefly comment on images we may acquire in these two cases. In the case where there is a fixed transverse magnetization in each repetition to start with, the image may display less SNR than the total signal optimized case. However, the constant signal guarantees high fidelity due to eliminated distortion in the phase encoding dimension. In contrast, in the case of maximum total magnetization, image distortion cannot be avoided but the image SNR is optimal.

Constant flip angle—In spite of the stable magnetization and high cumulative signal that is brought by variable flip angles, it posts technical challenges on MRI facilities to implement different flip angles in each repetition. A more widely used case is the constant flip angle, where the excitation pulses remain the same for all of the repetitions. We consider such case in this section and optimize the cumulative signal under such scenario.

The recurrent dynamic equation is similar despite the fact that θ is constant:

(11) $$\left\{\begin{array}{c} K_{n}=\left(K_{n-1}\cos\theta -K_{0}\right)e^{-\frac{TR}{T_{1}}}+K_{0}\\ K_{x,n}=K_{n}\sin\theta \end{array}\right.$$

Solving the recurrent dynamic equation:

(12) $$K_{x,n}=\pm K_{\mathrm{hp}}{\left(\Gamma \cos\theta \right)}^{n-1}\sin\theta +K_{0}\left(1-\Gamma \right)\sum_{k=1}^{n-1}{\left(\Gamma \cos\theta \right)}^{k-1}\sin\theta$$

**Figure A1:**
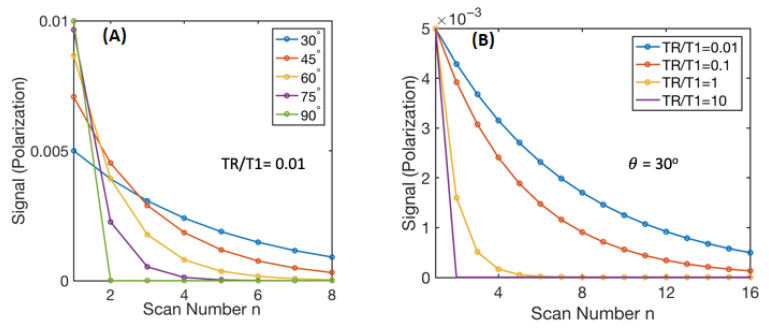
Simulation of signal of each individual repetition as a function of scan number of given TR/T1 in (**A**) and given θ in (**B**). In the simulation, we assume $K_{\mathrm{hp}}=10^{-3}$ and $K_{0}=10^{-5}$, which approximates the magnetization with our diamond imaging case at 9.4T.

Simulating this process, we observe the change of the magnetization with respect to *n* given a certain θ and TR/T1 in Figure A1. Note that in this case, we do not ignore the first term in Equation (12) because constant flip angle can lead to comparable magnitude of the first term with the second term.

**Figure 3:**
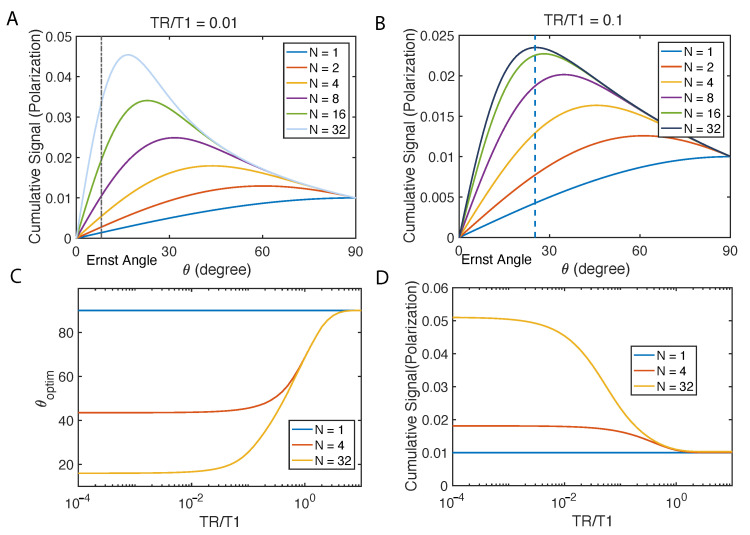
Constant flip angle. In the simulation, $K_{\mathrm{hp}}=10^{-3}$ and $K_{0}=10^{-5}$, which is on the same orders of magnitude of magnetization with our diamond imaging case at 9.4 T. (**A**) Cumulative signal with different total repetitions *N* is displayed when the ratio of TR/T1 is fixed. The black dash line is the Ernst angle, optimal for initial magnetization to be $M_{0}$. (**B**) Fixing the ratio TR/T1, we simulate the cumulative signal with different *N*. (**C**) Optimal flip angles and (**D**) resultant cumulative signals when such angle is restricted to a constant are shown as a function of TR/T1.

Similarly, we calculate cumulative signal: $S_{\mathrm{cumulative}\;}=\sum_{n=1}^{N}M_{x,n}$ in Figure 3. Not surprisingly, there is an optimal flip angle given certain TR/T1 and total number of scans *N*. Under such optimal angle, the case of N=32 displays a more than 4 times higher cumulative signal than 90${}^{\circ }$ pulse could (Figure 3A). When TR/T1 is less, increasing scan counts may become very effective for signal enhancement (Figure 3B). We compare this optimal flip angle with Ernst angle which is the flip angle for excitation of a particular spin that gives the maximal signal intensity in the least amount of time in the thermal polarization cases. We find that the optimal flip angle deviates from Ernst angle, however, approaching it when *N* increases.

It is difficult to optimize $S_{\mathrm{cumulative}\;}$ analytically, and we use a gradient descent method to numerically solve the problem, and the result is shown in Figure 3C,D.

### 2.3. Gradient Consideration

Gradient arrangement is another critical component in hyperpolarized solid state imaging. This determines timing for signal acquisition and k-space sampling trajectory and ultimately dictates image SNR, fidelity as well as resolution. Here we consider three categories of gradient arrangement, i.e. spin echo, gradient echo, and more exotic sequences. By analyzing different types of sequences, we provide insight into the gradient arrangement and sequence parameter determination for a given sample.

**Figure 4:**
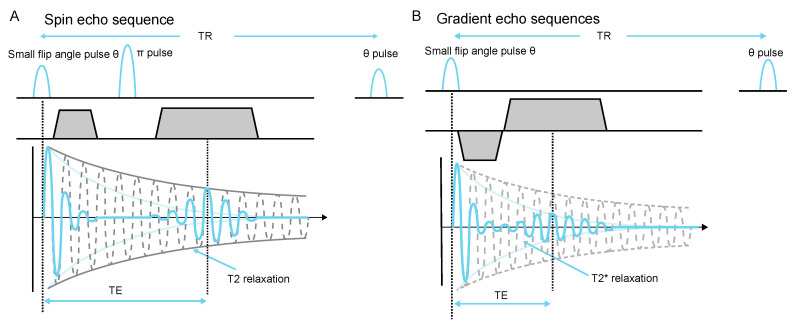
Spin echo and gradient echo sequence with small flip angle. (**A**) The π pulse refocuses dephasing caused by field inhomogeneity, chemical shift, and gradients. (**B**) The reversed gradient refocuses the effect of gradients. The phase encoding dimension implements same gradient arrangement for both of the two echo sequences, and is omitted here.

A typical spin echo sequence with small flip angle is shown in Figure 4. For a certain voxel (x,y,z), we consider the signal at the peak of the echo S(TE), which is a good indication of the image SNR. This signal within one voxel is subject to decoherence posterior to the flip angle pulse, and the decay factor is $e^{-TE_{se}/T_{2}}$ (see Figure 4A), where $TE_{se}$ is the echo time of a spin echo sequence. Similarly, in a gradient echo sequence, this factor becomes $e^{-TE_{ge}/T_{2}^{*}}$ (see Figure 4B). When assuming that both sequences use the same flip angle strategy, the decay factors imply that if $TE_{se}/T_{2}<TE_{ge}/T_{2}^{*}$, the spin echo sequence is favorable for higher signal; otherwise, one should choose gradient echo given that the signal at the peak of the echo is higher in such cases.

The RARE (Rapid Acquisition with Refocused Echoes) sequence, also known as TSE (turbo spin echo) is a sequence which takes advantage of multiple spin echo train followed by a single π/2 pulse. This sequence is originally designed for saturation recovery, can however be implemented with small flip angle excitation pulses, which may be enhance SNR in hyperpolarized solid state imaging. In this case, there can be T echo trains following a small flip angle excitation in each repetition.The cumulative signal depends on

(13) $$\sum_{j=1}^{N}\sum_{k=1}^{T}e^{-k\cdot TE_{se}/T_{2}}$$

where two summations of *j* and *k* represent repetitions and echo trains respectively. Carefully selection of *N* and *T* can possibly enhance the cumulative signal further than conventional spin echo or gradient echo sequences.

The sequences that decouple nuclear spins in solids, which we refer to here as “exotic sequences”, include magic echo sequence [18], as well as quadratic echo sequence [19]. However, those sequences are challenging to calibrate and implement due to the precise requirement of the spacing between pulses and the phase of the pulses.

Apart from forming spin echo or gradient echo, one can design the gradient arrangement so that signal acquisition can start immediately after the excitation pulse. For instance, steady gradient on both phase encoding and frequency encoding dimensions can be applied while the acquisition channel opens right after RF excitation, which corresponds to a radial trajectory in k-space. Such sequences are normally called Ultrashort TE, or UTE sequences [20]. Such methods can eliminate the decoherence happening before echo formation, although may have disadvantages in motion and gradient imperfection robustness [21].

### 2.4. Hyperpolarized Diamond Imaging Results

**Figure 5:**
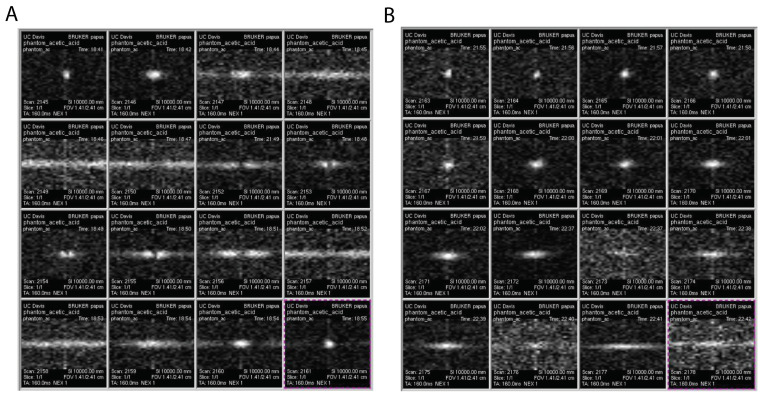
Diamond MRI with different flip angles. (**A**) The pulse durations are 5, 10, 15, ..., 80 μs respectively for each image. We can determine that optimal pulse duration should be within 20 μs. (**B**) The pulse durations are 4, 5, 6, ..., 19 μs respectively for each image. The text on each image is the frame number, time when the images are taken, and FOV (1.41 × 2.41 cm).

We test the above simulations using our hyperpolarized diamond imaging system [11]. A 5mm NMR tube is filled with diamond particles (average particle size ~200 μm) and the particles are tightly held at the bottom of the tube. The MRI images of such phantoms are shown in Figure 5 with different flip angles. We acquired images with flip angles ranging from 13–333${}^{\circ }$ by varying pulse length from 5 μs to 80 μs in Figure 5A, and we zoom in in the range of 4 μs to 19 μs to identify the optimal flip angle in Figure 5B. It turns out that the 6 μs presents the highest image fidelity and contrast. This shows agreement with Figure 3C, in which diamond particle imaging residents at low TR/T1 limit. Our diamond particles have a measured $T_{1}$ of 15 s and a repetition time TR of 6 ms for imaging, leading to TR/T1 $\sim 10^{-3}$, and corresponding $\theta_{\mathrm{optim}}$ of 16${}^{\circ }$. Such flip angle can be translated as a predicted 5.5 μs pulse length. Note that according to our nutation calibration, the pulse duration $t_{\mathrm{tip}}=\frac{\theta }{360^{\circ }}\times 84.58\;\mathrm{\mu s}+1.73\;\mathrm{\mu s}$, indicating a 1.73 μs delay of the pulse application by the MRI machine.

**Figure 6:**
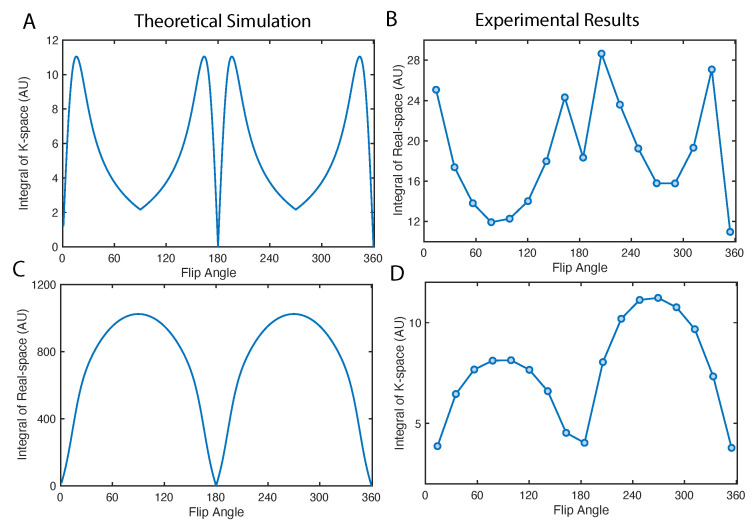
Total signal in k- and real- space as a function of flip angle. (**A**,**B**) The integration of absolute value in k-space is simulated and measured using the diamond particle phantom. (**C**,**D**) Display the integration of absolute value in real-space. The simulations are conducted assuming a uniform profile in real-space, i.e., ρ(x,y)= constant, in which case only the effect of magnetization factor *K* is emphasized.

We can study the total signal in k-space and real-space by taking the integral of intensities across all the pixels, shown in Figure 6. The k-space signal maximizes at the optimal flip angle in Figure 6A,B. Note that, a 90${}^{\circ }$ pulse can maximize the intensity of the center of the k-space, which is equivalent to the integral of real-space intensities (see Figure 6C,D). However such image has no high frequency information, which will be a constant in real-space along x direction. Such effect is precisely illustrated in Figure 5A first image in the second row.

## 3. Discussion

In the image equation (Equation (3)), the spin density function ρ(x,y) convolutes with $F(e^{-t\left(k_{y}\right)/T_{2}^{*}})$ and $F(K_{x}\left(k_{x}\right))$. The two terms correspond to two types of blur of the image. The term $F(e^{-t\left(k_{y}\right)/T_{2}^{*}})$ caused by $T_{2}$ is similar to the linewidth in NMR spectroscopy. The Lorentzian profile leads to a resolution limit of $\propto \frac{1}{\gamma GT_{2}}$ in real-space, where *G* is imaging gradient. The second term is a Fourier transform of the profile of magnetization as a function of repetition (see Appendix B Figure A1), originating from the uniformity of the magnetization distribution over repetitions. The term will reduce to 1 when flip angles in Figure 2A is applied. In our experiment, the two types of blur happen on x and y direction respectively. Our phase encoding is on x direction, therefore, the stripe line in Figure 5 originates from the Fourier transform of the magnetization factor profile *K* along $k_{x}$ direction. We write down $K_{x}\left(k_{x}\right)=\pm K_{\mathrm{hp}}{\left(\Gamma \cos\theta \right)}^{k_{x}-1}\sin\theta +K_{0}\left(1-\Gamma \right)\sum_{j=1}^{k_{x}-1}{\left(\Gamma \cos\theta \right)}^{j-1}\sin\theta$. If we take the 5th frame in Figure 5A as an example, the flip angle of that is close to 90${}^{\circ }$. The magnetization of such pulse sequence distributes mainly on the first repetition (green line in Appendix B Figure A1). A nearly constant $F(K_{x}\left(k_{x}\right))$ indicates the extreme case of blur—constant intensity along x direction when convoluting with ρ(x,y).

**Figure 7:**
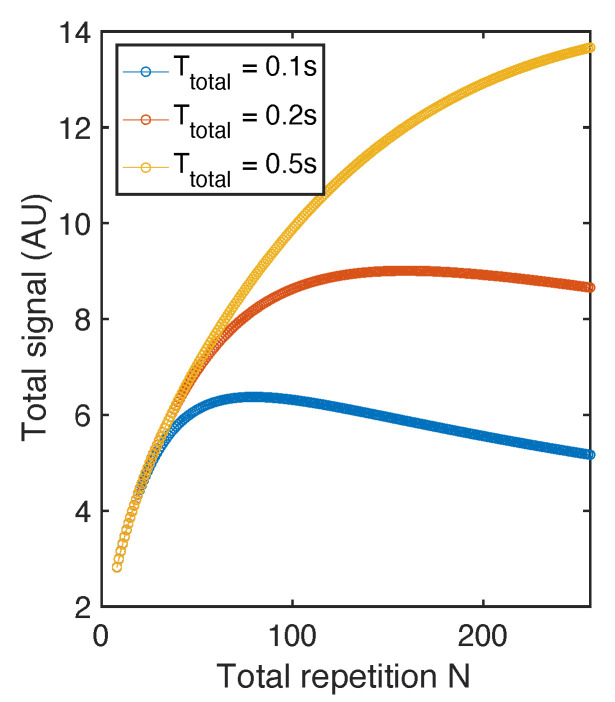
Total signal with RARE sequence. The simulation is conducted based on parameters close to diamonds ($T_{1}=$50 s, $T_{2}$ = 1 ms, TE= 0.5 ms). The total signal curve S(N) maximize at different *N* when $t_{total}$ is set to different values.

We would also like to discuss the total signal gained by the small tip angle RARE sequence. From Equation (13), we can tell that increasing number of echo trains will increase the signal, however extends the total acquisition times at the same time. Here we try to determine the optimal sequence design to maximize the total signal given a finite total time $T_{total}$. We take the case where one is allowed to vary the flip angle, and the signal is constant in each repetition (as described in Equation (7)). We assume that in each repetition $TR=TE_{se}\times T$, where *T* is total number of echos within this repetition. We can rewrite Equation (13) to estimate total signal as a function of total repetition number S(N):

(14) $$\begin{array}{cc} S(N) & =\sum_{j=1}^{N}\sum_{k=1}^{T(N)}e^{-k\cdot TE_{se}/T_{2}}\\ \; & =N\cdot M_{x}\left(N\right)\cdot \chi \frac{1-\chi^{T}}{1-\chi } \end{array}$$

where $\chi =e^{TE_{se}/T_{2}}$ is a constant when minimized TE is set by instrumentation limit and $T_{2}$ is the intrinsic property of certain sample. The above derivation used the sum of a geometric sequence. In this equation $M_{x}=M_{0}\times \sin\left(\theta_{1}\right)$ where $\theta_{1}$ defined in Equation (7) is a function of TR, and $TR=\frac{T_{total}}{N}$. *T* can also be written as a function of *N*: $T\left(N\right)=\frac{T_{total}}{N\cdot TE_{se}}$. We plot S(N) in Figure 7. We note that *N* values that can maximize S(N) for $T_{total}=$ 0.1 s and 0.2 s are ~70 and ~120 respectively. And when $T_{total}$ is long enough (0.5 s), S(N) is not yet saturated at N= 256.

## 4. Materials and Methods

### 4.1. Simulation and Optimization

The simulations and optimizations are conducted in Matlab, where the “fminunc” function is used to numerically optimize the flip angles in Figure 3C,D. In the simulation of Figure 6, the $K\left(k_{x}\right)e^{-t\left(k_{x},k_{y}\right)/T_{2}^{*}}$ component is simulated with $K(k_{x})$ in Equation (12), substituting *n* with $k_{x}$.

### 4.2. Hyperpolarization and Imaging

The diamond powder utilized in experiments in Figure 1 has ~40 mg mass with natural abundance ${}^{13}$C. The particles are purchased from Element6. They are enriched with ~1 ppm NV centers and fabricated by a high pressure high temperature (HPHT) protocol. The particle size is measured in SEM (scanning electron microscopy) images. The face to face distances are 200 μm to 250 μm and diagonal edge to edge distances are approximately 400 μm.

The entire experimental setup consists of three parts: a pneumatic field-cycling device, a wide-bore 9.4T superconducting magnet, an a miniaturized hyperpolarizer [22]. The pneumatic field-cycling device [11] is uses air flow to rapidly transfer a 5mm NMR tube from low field (40 mT) to the 9.4 T detection field, within which a 10 mm ${}^{1}$H/${}^{13}$C volume coil is installed. The air driven by a pump flows in a quartz channel and moves the NMR tube in the channel. Diamond samples are contained in the NMR tube. A concave-shaped stopper is located at the bottom end of the channel and a rubber stopper is placed at the high field. The transport time of the sample to high field is under 1s, much shorter compared to the ${}^{13}$C $T_{1}$ times (normally on the order of minitues). MR imaging was conducted with a Bruker DRX system equipped with microgradients running ParaVision 4 software with a modified FLASH pulse sequence. The miniaturized hyperpolarizer is a self-contained unit, which encapsulates devices for laser excitation, MW irradiation as well as an electromagnet for field fine-tuning. A 1W 520 nm diode laser (Lasertack PD-01289) is employed and the beam passes through an aspheric lens and a set of anamorphic prisms to form a 4 mm diameter beam. The beam was guided by two mirrors and illuminates the sample from the bottom. MW irradiation that drives polarization transfer is generated by three voltage controlled oscillator (VCO) sources (Minicircuits ZX95-3800A+). For frequency sweeps, the VCOs are driven by phase shifted triangle waves from a home-built PIC microprocessor (PIC30F2020) driven quad ramp generator.

Please find more details of experimental methods in Ref. [11].

## 5. Conclusions

In this paper, we studied two major components—small flip angles and gradient arrangement in a MRI sequence in the quest for optimal sequences for hyperpolarized solids. Both variable and constant flip angles are analyzed, and strategies to achieve maximum cumulative signal or flat signal profile are provided. Beyond designing flip angle progressions to take advantage of the significant initial magnetization produced by hyperpolarization, we propose to combine these excitation pulse progressions with traditional gradient arrangements in spin echo and gradient echo sequences in order to accommodate short decoherence times in solids. Experimental results of hyperpolarized diamond MRI show agreement with theoretical analysis. Beyond diamond particles, this study can provide guidance in hyperpolarized solids MRI in systems such as such as silicon [23] and silicon carbide [24] particles.

## Data Availability

The data presented in this study are available on request from the corresponding author.

## Appendix A. Derivation

### Appendix A.1. Variable Flip Angle for Constant Magnetization

(A1) $$K_{x,n}=K_{\mathrm{hp}}\left[\prod_{k=1}^{n-1}\left(\Gamma \cos\theta_{k}\right)\right]\sin\theta_{n}=\;\mathrm{constant}\;$$

Since we want to saturate the magnetization at the last pulse, we have $\sin\theta_{N}=1$. Using such equation, we can first write down the $K_{x,N}=K_{x,N-1}$ as:

(A2) $$K_{\mathrm{hp}}\left[\prod_{k=1}^{N-1}\left(\Gamma \cos\theta_{k}\right)\right]\sin\theta_{N}=K_{\mathrm{hp}}\left[\prod_{k=1}^{N-2}\left(\Gamma \cos\theta_{k}\right)\right]\sin\theta_{N-1}$$

This implies $\Gamma \cos\theta_{N-1}\sin\theta_{N}=\sin\theta_{N-1}$, and we can get: $\tan\theta_{N-1}=\sin\theta_{N}/\Gamma =1/\Gamma$. Similarly, if we take the equality between $K_{x,j}$ and $K_{x,j-1}$, the recursion formula is:

(A3) $$\tan\theta_{j-1}=\Gamma \sin\theta_{j}$$

Then, we need to solve $\theta_{n}$ based on the equation above. We define $a_{n}=\tan^{2}\theta_{n}$, and we will have:

(A4) $$a_{n}=\frac{a_{n+1}}{1+a_{n+1}}\cdot \Gamma^{2}$$

This is equivalent to:

(A5) $$\frac{1}{a_{n}}=\left(\frac{1}{a_{n+1}}-\Gamma^{2}\right)\Gamma^{2}$$

Solving the series, we can get:

(A6) $$a_{n}=\left(1-\Gamma^{3}\right)\cdot \frac{\Gamma^{2N-2n-1}}{1-\Gamma^{2N-2n-1}}$$

which leads to:

(A7) $$\tan^{2}\theta_{n}=\left(1-\Gamma^{3}\right)\cdot \frac{\Gamma^{2N-2n-1}}{1-\Gamma^{2N-2n-1}}$$

### Appendix A.2. Variable Flip Angle for Maximum Cumulative Magnetization

With

(A8) $$\Gamma \tan\theta_{j-1}=\sin\theta_{j}$$

We define $a_{n}=\tan^{2}\theta_{n}$, and we will have:

(A9) $$a_{n}=\frac{a_{n+1}}{1+a_{n+1}}\cdot \frac{1}{\Gamma^{2}}$$

This is equivalent to:

(A10) $$\frac{1}{a_{n}}=\left(\frac{1}{a_{n+1}}-\Gamma^{2}\right)/\Gamma^{2}$$

Solving the series, we can get:

(A11) $$a_{n}=\frac{1}{\Gamma^{2}}\cdot \frac{1-\Gamma^{2}}{1-\Gamma^{2(N-n)}}$$

which leads to:

(A12) $$\tan^{2}\theta_{n}=\frac{1}{\Gamma^{2}}\cdot \frac{1-\Gamma^{2}}{1-\Gamma^{2(N-n)}}$$

## Appendix B. Magnetization Simulation

The magnetization of each repetition when applying constant flip angle is presented.
